# Thalidomide dose selection for prevention of camrelizumab-induced reactive cutaneous capillary endothelial proliferation: a multicenter, randomized phase II trial

**DOI:** 10.3389/fimmu.2026.1917046

**Published:** 2026-10-16

**Authors:** Xiao Fu, Shuning Xu, Peng Cheng, Haitao Lan, Yina Wang, Dong Zhao, Mingyue Liu, Shouguo Li, Weidong Liu, Guiping Yu, Xiaowei Wei, Feilong Sun, Yu Yao, Ying Liu

**Affiliations:** 1 Department of Medical Oncology, The First Affiliated Hospital of Xi’an Jiaotong University, Xi’an, China; 2 Department of Digestive Diseases, Henan Cancer Hospital Affiliated Cancer Hospital of Zhengzhou University, Zhengzhou, China; 3 Department of Oncology, The First Affiliated Hospital of Nanyang Medical College, Nanyang, China; 4 Department of Oncology, Sichuan Academy of Medical Sciences/Sichuan Provincial People’s Hospital, Chengdu, China; 5 Medical Oncology Department, The First Affiliated Hospital of Medical School of Zhejiang University, Hangzhou, China; 6 Oncology Department, Lixin County People’s Hospital, Bozhou, China; 7 Medical Oncology Department, Henan Provincial People’s Hospital, Zhengzhou, China; 8 Radiation Oncology, Zhongshan Hospital Xiamen University, Xiamen, China; 9 Medical Oncology Department, Tangshan Central Hospital, Tangshan, China; 10 Medical Oncology Department, Xi’an NO. 9 Hospital, Xi’an, China; 11 Medical Oncology Department, Nanjing First Hospital, Nanjing, China; 12 Department of Medical Affairs, Jiangsu Hengrui Pharmaceuticals, Shanghai, China

**Keywords:** camrelizumab, esophageal squamous cell carcinoma, non-small cell lung cancer, reactive cutaneous capillary endothelial proliferation, thalidomide

## Abstract

**Background:**

Reactive cutaneous capillary endothelial proliferation (RCCEP) is a characteristic cutaneous adverse event associated with immune checkpoint inhibitors. This study compared two prophylactic thalidomide doses to identify the more promising dose for further evaluation.

**Methods:**

In this multicenter, randomized clinical trial, patients with advanced esophageal squamous cell carcinoma or non-small cell lung cancer (NSCLC) were randomly assigned to receive thalidomide 50 mg/day (Group A) or 100 mg/day (Group B) during first-line treatment with camrelizumab plus standard chemotherapy. The primary endpoint was overall RCCEP incidence; secondary endpoints included grade ≥3 RCCEP, median time to RCCEP onset, week 6 and week 9 RCCEP incidence, and safety.

**Results:**

A total of 72 patients were randomized. RCCEP occurred in 12 of 36 patients (33.3%) in Group A and 9 of 36 patients (25.0%) in Group B. RCCEP incidence at week 6 was 13.9% in Group A and 19.4% in Group B, and by week 9 was 25.0% and 19.4%, respectively. No grade ≥3 RCCEP occurred; all first RCCEP events were grade 1, with 1 grade 2 event reported in Group A. Among patients who developed RCCEP, median onset time was 1.49 and 1.31 months, respectively. Thalidomide-related adverse events occurred in 22.2% and 41.7% of patients, and grade 3 or higher thalidomide-related adverse events occurred in 8.3% and 22.2%, respectively. Serious adverse events occurred in 11.1% and 13.9% of patients, respectively.

**Conclusion:**

Thalidomide 100 mg/day met the prespecified incidence-based dose-selection criterion compared with 50 mg/day but was associated with a higher incidence of grade 3 or higher toxicity. Further controlled evaluation is warranted.

**Clinical trial registration:**

https://clinicaltrials.gov/study/NCT06231680, identifier NCT06231680.

## Introduction

Immune checkpoint inhibitors restore antitumor T-cell immunity by blocking the immunosuppressive signaling mediated by programmed death 1 (PD-1) and its ligand PD-L1 (1, 2). In recent years, PD-1/PD-L1 inhibitors have become an integral component of systemic therapy for multiple solid tumors and have demonstrated clear clinical benefits in lung cancer, esophageal cancer, hepatocellular carcinoma, and other malignancies (3–5). With the expanding use of immunotherapy, immune-related adverse events (irAEs) have emerged as a major clinical concern. irAEs can involve multiple organ systems, including the skin, gastrointestinal tract, endocrine organs, lungs, and liver, among which cutaneous toxicity is one of the most common categories. Although most cutaneous irAEs are low grade, they may still compromise quality of life and lead to treatment delay or modification (6, 7).

Camrelizumab is a humanized IgG4 monoclonal antibody against PD-1 that restores T-cell–mediated antitumor activity by blocking the interaction between PD-1 and PD-L1 (8). Previous studies have shown that camrelizumab has antitumor activity across multiple solid tumors with a manageable safety profile (9). Compared with other PD-1 inhibitors, camrelizumab is associated with a distinctive cutaneous irAE, reactive cutaneous capillary endothelial proliferation (RCCEP), which has been reported in multiple studies as one of the most common adverse events during camrelizumab therapy and has been correlated with antitumor response (10–12).

Clinically, RCCEP typically presents as red papules or nodular lesions that may be solitary or multifocal; ulceration or bleeding can occur in a subset of patients. Histopathological examination of RCCEP lesions is characterized predominantly by proliferation of capillary-like structures in the dermis accompanied by endothelial cell hyperplasia (13). The mechanisms underlying RCCEP have not been fully elucidated. Notably, cohort studies have suggested an association between antiangiogenic combination therapy and a reduced risk of RCCEP. In a retrospective study of non-small cell lung cancer (NSCLC), camrelizumab combined with antiangiogenic agents was associated with a lower incidence of RCCEP (14, 15). Similarly, early studies of camrelizumab plus apatinib reported encouraging efficacy with a manageable safety profile (16). Thalidomide has antiangiogenic properties and was shown to inhibit angiogenesis in a seminal study (17). For RCCEP prevention, a prospective study evaluated thalidomide prophylaxis for camrelizumab-induced RCCEP and reported a reduced RCCEP incidence with acceptable overall safety (18).

On the basis of this evidence, we conducted a multicenter randomized clinical trial to investigate the efficacy and safety of thalidomide for preventing RCCEP during first-line camrelizumab plus standard chemotherapy, and to compare the preventive effects and tolerability of two doses (50 mg/day vs. 100 mg/day). We aimed to provide evidence to support prophylactic dose selection for the management of camrelizumab-associated RCCEP.

## Methods

### Study design and patients

This was a prospective, multicenter, randomized phase II selection trial and represents the final analysis of the prevention cohort of the parent study registered at ClinicalTrials.gov (NCT06231680). The treatment cohort enrolled 19 patients but was terminated early because thalidomide showed insufficient therapeutic activity for established RCCEP. No formal interim analysis was performed, and only the prevention cohort is reported here.

Patients were recruited from 11 centers, including the First Affiliated Hospital of Xi’an Jiaotong University and Henan Cancer Hospital, between January 2024 and March 2025. The study was conducted in accordance with the Declaration of Helsinki and Good Clinical Practice guidelines. The study protocol and all amendments were approved by the ethics committees of all 11 participating centers before patient enrollment. Written informed consent was obtained from all patients before treatment initiation.

Eligible patients were adults aged 18 years or older with histologically or cytologically confirmed advanced NSCLC or esophageal squamous cell carcinoma (ESCC). Patients were required to have received no prior systemic therapy, be scheduled to receive first-line camrelizumab plus platinum-based doublet chemotherapy, have an Eastern Cooperative Oncology Group performance status of 0 to 1, and an expected survival of at least 12 weeks. Additional eligibility criteria included no previous treatment with immune checkpoint inhibitors and adequate organ and bone marrow function.

Key exclusion criteria included hypersensitivity to study drugs, recent immunosuppressive therapy, active autoimmune disease or infection, uncontrolled central nervous system metastases, clinically significant pulmonary disease, recent major cardiovascular or cerebrovascular events, and other conditions that could interfere with safety assessment.

### Procedures

Patients were randomly assigned to Group A or Group B using block randomization with a 1:1 allocation ratio and no additional stratification factors. The random allocation sequence was generated and maintained by an independent third party and was not accessible to investigators or study personnel before treatment assignment. Both groups received first-line camrelizumab plus platinum-based doublet chemotherapy. Camrelizumab was administered at a fixed dose of 200 mg every 3 weeks. Patients in Group A and Group B received oral thalidomide at doses of 50 mg once daily and 100 mg once daily, respectively, until 4 weeks after the last dose of camrelizumab. The chemotherapy regimen was selected according to tumor type using a standard platinum-based doublet regimen. Thalidomide was initiated on the same day as the first dose of camrelizumab. Temporary treatment interruption and protocol-defined dose reductions were permitted for treatment-related toxicity. In Group A, thalidomide could be reduced once from 50 to 25 mg/day; in Group B, up to three stepwise reductions from 100 to 75, 50, and 25 mg/day were permitted. Dose re-escalation was not allowed. Adherence was monitored through study-drug accountability records. After RCCEP onset, management followed the 2023 CSCO guideline for immune checkpoint inhibitor-related toxicities; no cryotherapy or laser treatment was used.

After 4 to 6 cycles of combination therapy, patients entered the maintenance phase. Patients with ESCC or squamous NSCLC received camrelizumab monotherapy as maintenance treatment, whereas patients with non-squamous NSCLC received camrelizumab plus pemetrexed maintenance therapy. Treatment was continued until disease progression, unacceptable toxicity, or completion of 2 years of camrelizumab treatment.

### Outcomes

The primary endpoint was the incidence of RCCEP. Secondary endpoints included the incidence of grade 3 or higher RCCEP, median time to RCCEP onset, RCCEP incidence at week 6, RCCEP incidence at week 9, and thalidomide-related adverse events. For the prevention cohort, overall RCCEP incidence was assessed throughout the observation period, whereas week 6 and week 9 RCCEP incidences were prespecified landmark secondary endpoints. The 3-week RCCEP response endpoint registered for the parent study pertained to the treatment cohort and was not included in the present analysis. From the time of randomization, a comprehensive skin examination was performed every 3 weeks, including assessment of the body surface skin, periocular skin, eyelids, nasal mucosa, and oral mucosa.

No fixed risk window or specific final-analysis trigger for overall RCCEP incidence was prespecified. Overall RCCEP incidence was calculated using RCCEP events observed from treatment initiation through the data cutoff or the patient’s last available follow-up. For RCCEP episodes that had not resolved by the data cutoff, the last available follow-up date was used as the end date for calculating RCCEP duration.

The study was open-label, and RCCEP assessors were not masked. At each center, licensed physicians experienced in oncology clinical trials performed skin and mucosal examinations at screening, before each treatment cycle, and at study completion or withdrawal, with additional assessments for reported skin abnormalities. Investigators received RCCEP-specific training and used a study-specific observation form; lesions were photographed, and atypical cases were referred for dermatology consultation. No central adjudication was performed, and consistency across centers was supported by uniform criteria, investigator training, and photographic review. RCCEP severity was graded according to prespecified criteria based on the 2023 CSCO guideline for the management of immune checkpoint inhibitor-related toxicities (19). Grade 1 was defined by cutaneous and/or mucosal lesions with a maximum diameter of ≤10 mm, grade 2 by lesions >10 mm, grade 3 by generalized lesions potentially complicated by infection or requiring hospitalization, and grade 4 by life-threatening manifestations; grade 5 indicated death.

### Adverse events

All adverse events (AEs) occurring during the study, including clinical symptoms, abnormalities in vital signs, and laboratory abnormalities, were recorded with respect to their clinical characteristics, severity, onset time, end time, management, and outcome. The relationship between each adverse event and the study drugs was also assessed. Safety was evaluated according to the National Cancer Institute Common Terminology Criteria for Adverse Events, version 5.0.

### Quality of life

Patient-reported outcomes (PROs) were assessed using the European Organization for Research and Treatment of Cancer Quality of Life Questionnaire-Core 30 (EORTC QLQ-C30). Data were collected through self-administered electronic questionnaires at baseline, week 6, and the end of treatment. QoL analyses were exploratory. Changes from baseline were analyzed using a repeated-measures mixed-effects model, with treatment group, visit, treatment-by-visit interaction, and baseline value as fixed effects. Subject ID was treated as the subject effect, with visit specified as the repeated factor using an unstructured covariance matrix. The Kenward-Roger approximation was used for denominator degrees of freedom. Least-squares means (LSMs) and corresponding 95% confidence intervals were estimated for each treatment group at each visit.

### Statistical analysis

This study was designed as a randomized phase II selection trial to identify the more promising thalidomide dose for further evaluation. Sample size was determined according to the randomized phase II selection design described by Simon et al. (20), assuming RCCEP incidences of 40% in Group A and 30% in Group B (18, 21). A minimum of 34 patients per group provided at least an 80% probability of correctly selecting the regimen with the lower observed RCCEP incidence, irrespective of the magnitude of the observed between-group difference; allowing for an approximately 5% dropout rate, 36 patients per group were planned.

No formal superiority hypothesis test between the two dose groups was prespecified. According to the protocol, an absolute difference of more than 5 percentage points in overall RCCEP incidence was used as the selection threshold, with the regimen showing the lower incidence considered more promising. If the observed difference was 5 percentage points or less, the prespecified incidence-based selection threshold would not be met, and such a result would require cautious interpretation. No prespecified safety-based dose-selection criterion was used.

The intention-to-treat (ITT) population included all randomized patients. The full analysis set (FAS) and safety set (SS) included all patients who received at least 1 dose of study treatment and were used for efficacy and safety analyses, respectively. Continuous variables were summarized using the number of patients, mean, standard deviation, median, quartiles, minimum, and maximum. Categorical variables were summarized using frequencies and percentages.

Overall RCCEP incidence, grade 3 or higher RCCEP incidence, and RCCEP incidence at weeks 6 and 9 were analyzed in the FAS. The week 6 and week 9 analyses were prespecified landmark analyses using all patients in the FAS as the denominator; RCCEP status was evaluable for all patients at both timepoints, with no missing assessments. Two-sided 95% confidence intervals for RCCEP incidence were estimated using the Clopper-Pearson method. RCCEP onset time was analyzed descriptively only among patients who developed RCCEP and was defined as the interval from study treatment initiation to the first documented RCCEP. RCCEP duration was likewise analyzed descriptively as a continuous variable rather than as a time-to-event endpoint.

## Results

### Patients

#### Baseline characteristics

Between January 2024 and March 2025, a total of 72 patients were screened and all were enrolled and randomized, with 36 assigned to each treatment group. No screening failures or pre-treatment withdrawals were reported. All randomized patients received at least 1 dose of study treatment and were included in the FAS and SS populations. A total of 23 patients were evaluable for PRO analysis. At the data cutoff of November 26, 2025, the median follow-up was 6.5 months (IQR, 3.9-7.5) in Group A and 7.0 months (IQR, 5.0-8.9) in Group B. Thirteen patients remained on treatment, 4 patients had completed treatment, and 55 had discontinued treatment. Treatment discontinuation occurred in 28 patients in Group A and 27 patients in Group B. The corresponding reasons were patient request (13 vs 9), clinical progression (5 vs 7), withdrawal of consent (3 vs 2), adverse events (1 vs 3), investigator decision (2 vs 2), radiographic progression (0 vs 3), loss to follow-up (2 vs 0), and other reasons (2 vs 1), respectively (**Figure 1**).

**Figure 1:**
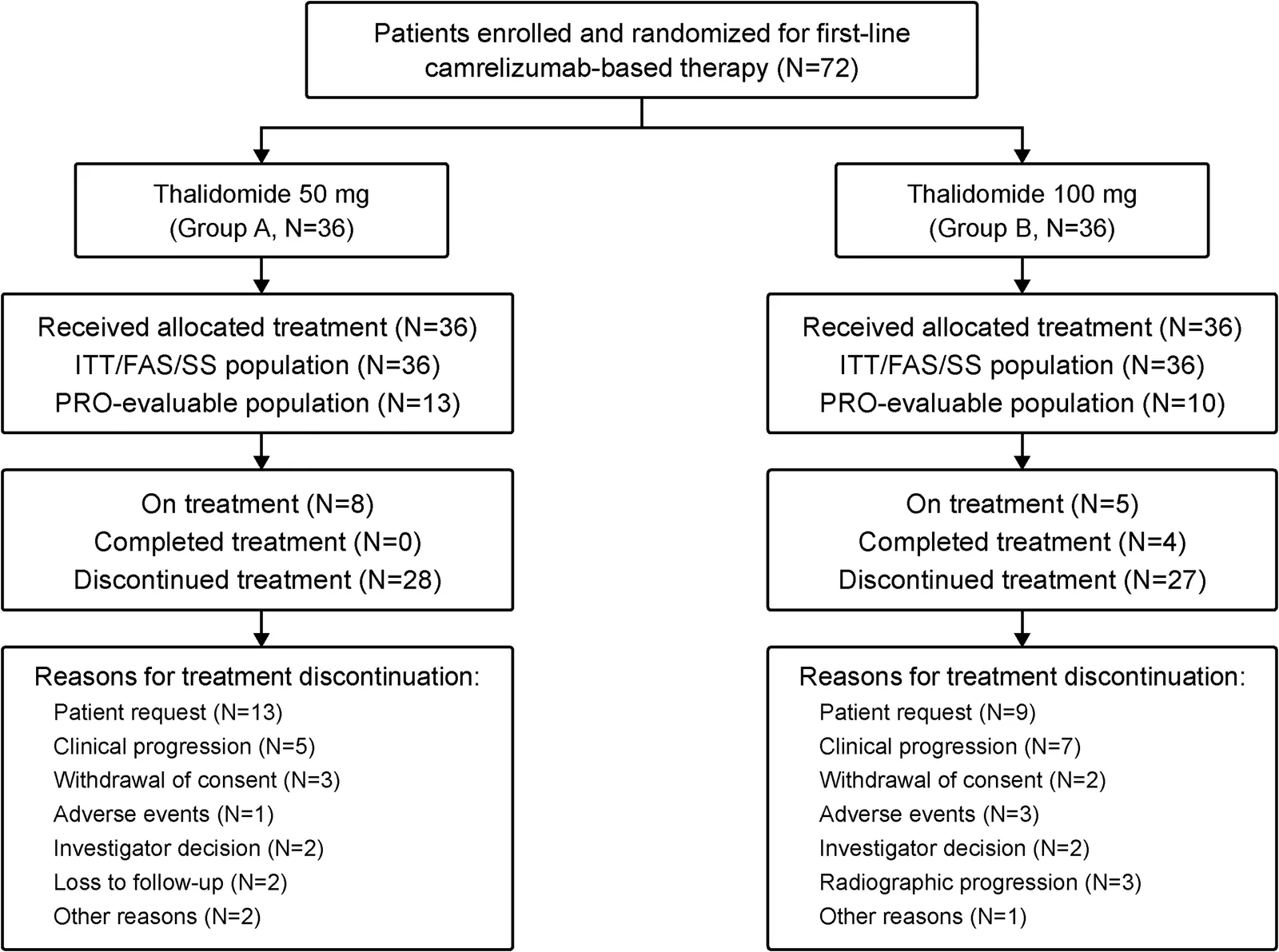
Patients’ flowchart.

Baseline characteristics were summarized in **Table 1**. The median age was 69.0 years in Group A and 66.5 years in Group B, most patients were male, and ECOG performance status was 1 in approximately two-thirds of patients. Lung cancer accounted for 44.4% and 61.1% of cases, and ESCC for 55.6% and 38.9%, respectively. All but one patient had stage III or IV disease at baseline. One patient with stage II ESCC was enrolled despite the protocol-defined advanced-disease eligibility criterion and was retained in the analysis populations after randomization and treatment initiation (**Table 1**).

**Table 1:** Baseline characteristics.

Demographics	Group A (N = 36)	Group B (N = 36)
Age (years)		
Median age (IQR)	69.0 (63.5, 73.0)	66.5 (60.0, 70.0)
≤ 65	10 (27.8)	16 (44.4)
> 65	26 (72.2)	20 (55.6)
Gender, n (%)		
Male	31 (86.1)	29 (80.6)
Female	5 (13.9)	7 (19.4)
BMI (kg/m^2^)		
Median (IQR)	21.58 (19.70, 23.53)	23.23 (20.86, 24.80)
ECOG, n (%)		
0	14 (38.9)	13 (36.1)
1	22 (61.1)	23 (63.9)
Tumor type, n (%)		
Lung cancer	16 (44.4)	22 (61.1)
Esophageal cancer	20 (55.6)	14 (38.9)
Tumor stage, n (%)		
Lung cancer	n=16	n=22
I	0	0
II	0	0
III	3 (18.8)	4 (18.2)
IV	13 (81.3)	18 (81.8)
Esophageal cancer	n=20	n=14
I	0	0
II	0	1 (7.1)
III	2 (10.0)	6 (42.9)
IV	18 (90.0)	7 (50.0)
Metastatic status, n (%)		
Lung cancer	n=16	n=22
Yes	13 (81.3)	18 (81.8)
No	3 (18.8)	4 (18.2)
Esophageal cancer	n=20	n=14
Yes	12 (60.0)	4 (28.6)
No	8 (40.0)	10 (71.4)

IQR, interquartile range; BMI, body mass index; ECOG, Eastern Cooperative Oncology Group; Percentages for tumor stage and metastatic status were calculated within each tumor subgroup. Metastatic status refers to the presence of distant metastasis. Percentages may not sum to 100% because of rounding.

### Treatment exposure

Exposure to camrelizumab and thalidomide was generally comparable between groups. Median relative dose intensity was 98.44% and 94.18% for camrelizumab, and 100.00% in both groups for thalidomide. Median adherence was 100.00% for both drugs in both groups (**Table 2**).

**Table 2:** Treatment exposure and relative dose intensity.

Drug	Parameter	Group A	Group B
Camrelizumab	Exposure duration, 21-day cycles, median (Q1, Q3)	3.88 (2.07, 12.43)	5.55 (2.64, 9.07)
	Administered cycles, median (Q1, Q3)	4.50 (2.50, 9.50)	5.00 (3.00, 8.00)
	Relative dose intensity, %, median (Q1, Q3)	98.44 (89.62, 100.00)	94.18 (86.18, 100.00)
	Adherence, %, median (Q1, Q3)	100.00 (100.00, 100.00)	100.00 (100.00, 100.00)
Thalidomide	Exposure duration, 21-day cycles, median (Q1, Q3)	3.90 (2.00, 11.21)	4.81 (2.33, 10.43)
	Administered cycles, median (Q1, Q3)	3.64 (2.00, 8.98)	4.00 (2.31, 9.62)
	Relative dose intensity, %, median (Q1, Q3)	100.00 (91.76, 100.00)	100.00 (97.70, 100.00)
	Adherence, %, median (Q1, Q3)	100.00 (91.76, 100.00)	100.00 (98.48, 100.00)

Exposure duration represents elapsed treatment time expressed in 21-day cycles and was calculated as [min (last dose date + 21 days, date of death) - first dose date]/21. Administered cycles were defined as cycles with a nonzero dose. Relative dose intensity was calculated as observed dose intensity divided by the protocol-specified dose intensity ×100, and adherence as total administered dose divided by total prescribed dose ×100.

### Efficacy

#### Overall

In the FAS, RCCEP occurred in 12 of 36 patients (33.3%; 95% CI, 18.6%-51.0%) in the thalidomide 50 mg group and in 9 of 36 patients (25.0%; 95% CI, 12.1%-42.2%) in the thalidomide 100 mg group. The absolute risk difference for Group A versus Group B was 8.3 percentage points (95% CI, -12.9 to 28.9), and the corresponding relative risk was 1.3 (95% CI, 0.6-2.8) (**Table 3**). The incidence of RCCEP by week 6 was 13.9% (5/36; 95% CI, 4.7%-29.5%) in the 50 mg group and 19.4% (7/36; 95% CI, 8.19%-36.02%) in the 100 mg group. By week 9, RCCEP had occurred in 9 of 36 patients (25.0%; 95% CI, 12.1%-42.2%) in the 50 mg group and 7 of 36 patients (19.4%; 95% CI, 8.2%-36.0%) in the 100 mg group. Among patients who developed RCCEP, the median time to RCCEP onset was 1.49 months (IQR, 1.00-2.15) in the 50 mg group and 1.31 months (IQR, 1.05-1.41) in the 100 mg group. The median total duration of RCCEP was 70.0 days (IQR, 34.5-130.0) and 44.0 days (IQR, 36.0-69.0), respectively. All first RCCEP events were grade 1 in both groups. According to the highest RCCEP grade observed during treatment, 11 patients (30.6%) in the 50 mg group and 9 patients (25.0%) in the 100 mg group had grade 1 RCCEP, while 1 patient (2.8%) in the 50 mg group experienced grade 2 RCCEP. No grade 3 or higher RCCEP was observed in either group (**Table 3**).

**Table 3:** Overall efficacy.

Items	Group A (N = 36)	Group B (N = 36)
Overall RCCEP incidence, n (%; 95% CI)	12 (33.3; 18.6-51.0)	9 (25.0; 12.1-42.2)
Risk difference, percentage points (95% CI), Group A minus Group B	8.3 (-12.9 to 28.9)	
Relative risk (95% CI), Group A vs Group B	1.3 (0.6 to 2.8)	
Median time to RCCEP onset (months)*		
Median (IQR)	1.49 (1.00, 2.15)	1.31 (1.05, 1.41)
Total duration of RCCEP, days		
Median (IQR)	70.0 (34.5, 130.0)	44.0 (36.0, 69.0)
RCCEP incidence by week 6, n (%; 95% CI)	5 (13.9; 4.7-29.5)	7 (19.4; 8.19-36.02)
RCCEP incidence by week 9, n (%; 95% CI)	9 (25.0; 12.1-42.2)	7 (19.4; 8.2-36.0)
Grade at first RCCEP occurrence, n (%)		
Grade 1	12 (33.3)	9 (25.0)
Grade 2	0	0
Grade 3	0	0
Grade 4	0	0
Grade 5	0	0
Highest RCCEP grade, n (%)		
Grade 1	11 (30.6)	9 (25.0)
Grade 2	1 (2.8)	0
Grade 3	0	0
Grade 4	0	0
Grade 5	0	0

The risk difference was calculated as Group A minus Group B, with the 95% CI estimated using the Miettinen-Nurminen method. Relative risk was calculated for Group A versus Group B, with the 95% CI estimated using the Wald method. ^*^Calculated among patients who developed RCCEP. RCCEP, reactive cutaneous capillary endothelial proliferation; IQR, interquartile range.

Among patients who developed RCCEP, trunk involvement was the most common lesion site in both groups, reported in 10 of 12 patients (83.3%) in the 50 mg group and 6 of 9 patients (66.7%) in the 100 mg group. Head and face involvement was observed in 7 of 12 patients (58.3%) and 3 of 9 patients (33.3%), respectively, whereas neck involvement was reported in 3 of 12 patients (25.0%) and 5 of 9 patients (55.6%). Red nevus-like lesions were the most common morphologic subtype in both groups, occurring in 9 of 12 patients (75.0%) in the 50 mg group and 6 of 9 patients (66.7%) in the 100 mg group. Bleeding was reported in 2 of 12 patients (16.7%) in the 50 mg group and in 1 of 9 patients (11.1%) in the 100 mg group. Most patients had fewer than 10 lesions, occurring in 7 of 12 patients (58.3%) in the 50 mg group and 5 of 9 patients (55.6%) in the 100 mg group. The median maximum lesion diameter was 4.5 mm (IQR, 3.5-6.0) in the 50 mg group and 3.5 mm (IQR, 2.0-5.0) in the 100 mg group (**Supplementary Table S1**).

### Subgroup

In the ESCC subgroup, RCCEP occurred in 6 of 20 patients (30.0%; 95% CI, 11.9%-54.3%) in the thalidomide 50 mg group and in 4 of 14 patients (28.6%; 95% CI, 8.4%-58.1%) in the thalidomide 100 mg group. The median total duration of RCCEP was 44.0 days (IQR, 22.0-168.0) and 33.0 days (IQR, 11.5-72.0), respectively. All first RCCEP events were grade 1 in both groups. According to the highest RCCEP grade observed during treatment, 5 patients (25.0%) in the 50 mg group and 4 patients (28.6%) in the 100 mg group had grade 1 RCCEP, while 1 patient (5.0%) in the 50 mg group had grade 2 RCCEP; no grade 2 or higher RCCEP was observed in the 100 mg group (**Table 4**).

**Table 4:** Efficacy endpoints in the ESCC subgroup.

Items	Group A (N = 20)	Group B (N = 14)
Overall RCCEP incidence, n (%; 95% CI)	6 (30.0; 11.9-54.3)	4 (28.6; 8.4-58.1)
Total duration of RCCEP, days		
Median, (IQR)	44.0 (22.0, 168.0)	33.0 (11.5, 72.0)
Grade at first RCCEP occurrence, n (%)		
Grade 1	6 (30.0)	4 (28.6)
Grade 2	0	0
Grade 3	0	0
Grade 4	0	0
Grade 5	0	0
Highest RCCEP grade, n (%)		
Grade 1	5(25.0)	4 (28.6)
Grade 2	1 (5.0)	0
Grade 3	0	0
Grade 4	0	0
Grade 5	0	0

ESCC, esophageal squamous carcinoma.

In the NSCLC subgroup, RCCEP occurred in 6 of 16 patients (37.5%; 95% CI, 15.2%-64.6%) in the thalidomide 50 mg group and in 5 of 22 patients (22.7%; 95% CI, 7.8%-45.4%) in the thalidomide 100 mg group. The median total duration of RCCEP was 72.5 days (IQR, 69.0-92.0) in the 50 mg group and 49.0 days (IQR, 41.0-69.0) in the 100 mg group. All first RCCEP events were grade 1 in both groups, and all events remained grade 1 at the highest observed severity in both groups (**Table 5**).

**Table 5:** Efficacy endpoints in the NSCLC subgroup.

Items	Group A (N = 16)	Group B (N = 22)
Overall RCCEP incidence, n (%; 95% CI)	6 (37.5; 15.2-64.6)	5 (22.7; 7.8-45.4)
Total duration of RCCEP, days		
Median, (IQR)	72.5 (69.0, 92.0)	49.0 (41.0, 69.0)
Grade at first RCCEP occurrence, n (%)		
Grade 1	6 (37.5)	5 (22.7)
Grade 2	0	0
Grade 3	0	0
Grade 4	0	0
Grade 5	0	0
Highest RCCEP grade, n (%)		
Grade 1	6 (37.5)	5 (22.7)
Grade 2	0	0
Grade 3	0	0
Grade 4	0	0
Grade 5	0	0

NSCLC, non-small cell lung cancer.

Detailed lesion characteristics, including lesion site, morphologic subtype, bleeding status, lesion count, and maximum diameter, are summarized separately for patients with ESCC and NSCLC in **Supplementary Table S2**, **S3**, respectively.

### AE

In the safety set, treatment-emergent adverse events (TEAEs) of any grade occurred in 27 of 36 patients (75.0%) in the thalidomide 50 mg group and 28 of 36 patients (77.8%) in the thalidomide 100 mg group. Grade 3 or higher TEAEs were reported in 6 patients (16.7%) and 13 patients (36.1%), respectively. Serious adverse events (SAEs) were reported in 4 of 36 patients (11.1%) in the 50 mg group and 5 of 36 patients (13.9%) in the 100 mg group (**Table 6**).

**Table 6:** Overall safety summary in the safety set.

Safety outcome	Group A (n=36)	Group B (n=36)
Any TEAE, n (%)	27 (75.0)	28 (77.8)
Grade ≥3 TEAE, n (%)	6 (16.7)	13 (36.1)
Any TRAE, n (%)	21 (58.3)	22 (61.1)
Grade ≥3 TRAE, n (%)	3 (8.3)	10 (27.8)
Any TESAE, n (%)	4 (11.1)	5 (13.9)

TEAE, treatment-emergent adverse event; TRAE, treatment-related adverse event; TESAE, treatment-emergent serious adverse event.

Thalidomide-related AEs of any grade occurred in 8 of 36 patients (22.2%) in the 50 mg group and 15 of 36 patients (41.7%) in the 100 mg group, while grade 3 or higher events occurred in 3 patients (8.3%) and 8 patients (22.2%), respectively. The most common thalidomide-related AEs were neutrophil count decreased (2.8% vs 13.9%), white blood cell count decreased (0 vs 13.9%), anemia (2.8% vs 11.1%), and asthenia (0 vs 8.3%). Additional events occurring in at least 5% of patients in either group included hypothyroidism, hyperthyroidism, lymphocyte count decreased, platelet count decreased, and dysphagia (**Table 7**).

**Table 7:** Thalidomide-related adverse events occurring in at least 5% of patients in either treatment group.

Events	Group A Any grade, n (%)	Group A Grade ≥3, n (%)	Group B Any grade, n (%)	Group B Grade ≥3, n (%)
Neutrophil count decreased	1 (2.8)	0	5 (13.9)	4 (11.1)
White blood cell count decreased	0	0	5 (13.9)	1 (2.8)
Anemia	1 (2.8)	0	4 (11.1)	0
Asthenia	0	0	3 (8.3)	1 (2.8)
Hypothyroidism	0	0	2 (5.6)	0
Hyperthyroidism	0	0	2 (5.6)	0
Lymphocyte count decreased	0	0	2 (5.6)	0
Dysphagia	2 (5.6)	1 (2.8)	0	0
Platelet count decreased	0	0	2 (5.6)	0

### QoL

In the PRO-evaluable population (n=23; 50 mg group, n=13; 100 mg group, n=10), exploratory QoL analyses showed numerical differences between groups in global health status/QoL and selected functional domains. At week 6, the LSM change in global health status/QoL was 11.8 in the 100 mg group versus -2.4 in the 50 mg group. At end of treatment (EOT), the LSM change in physical functioning was 5.3 versus -4.4, respectively. Given the limited evaluable population and variable timing of EOT assessments, these findings should be interpreted as highly exploratory (**Figure 2**).

**Figure 2:**
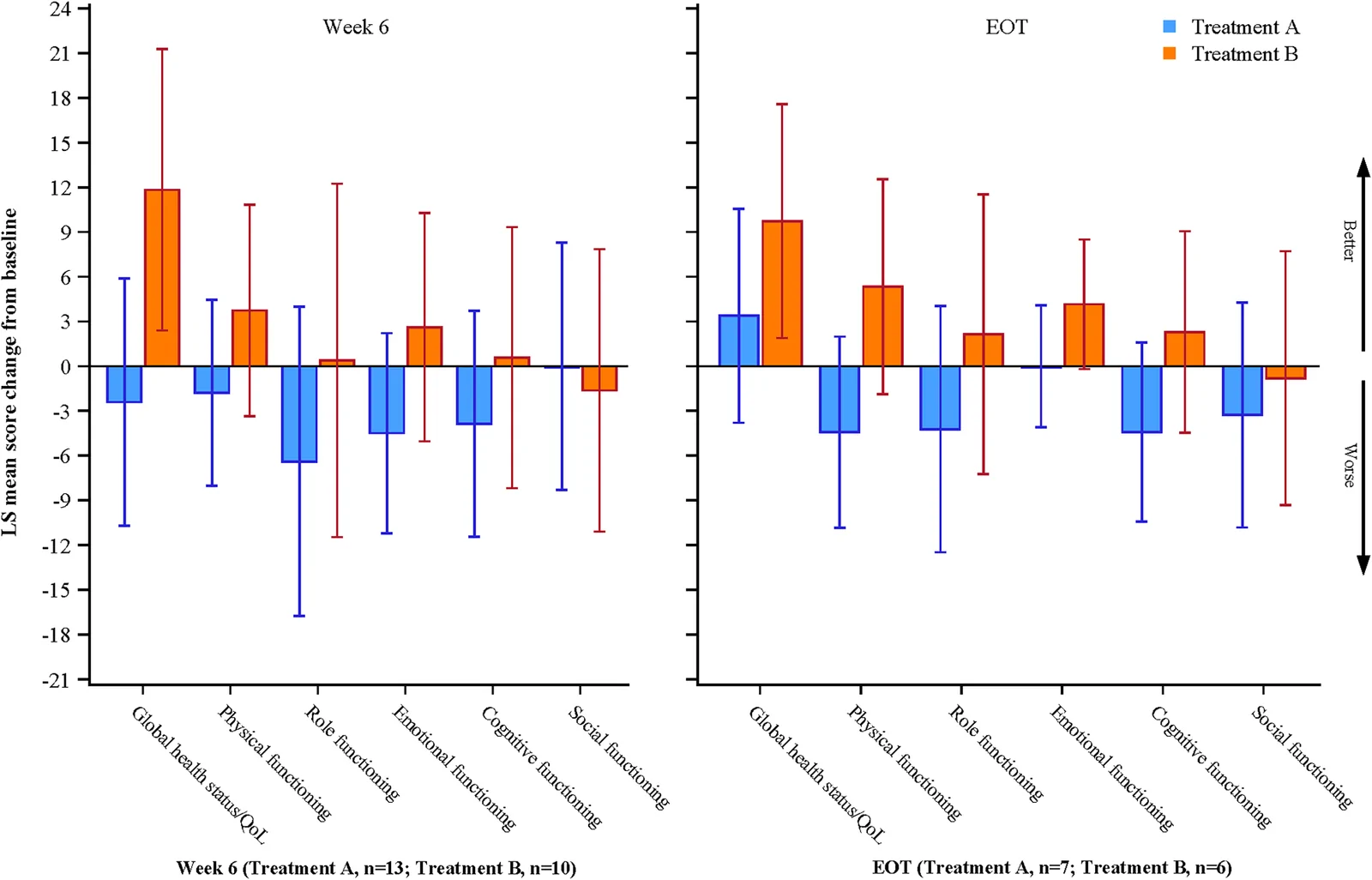
LSM changes from baseline in EORTC QLQ-C30 scores at week 6 and EOT. Error bars indicate 95% CIs. Treatment A, thalidomide 50 mg/day; Treatment B, thalidomide 100 mg/day.

## Discussion

In this multicenter, randomized phase II trial, thalidomide prophylaxis was associated with a relatively low overall incidence of RCCEP in both treatment groups, with numerically lower overall and week 9 RCCEP incidence in Group B than in Group A, and no grade 3 or higher RCCEP observed in either group. In addition, RCCEP in both groups was predominantly grade 1, and Group B showed numerically shorter RCCEP duration, although this was accompanied by a higher incidence of grade 3 or higher TEAEs and thalidomide-related AEs. Taken together, our findings support the selection of 100 mg/day as the more promising thalidomide dose for further evaluation of RCCEP prophylaxis during first-line camrelizumab plus chemotherapy, while highlighting the need to balance the numerical preventive advantage against the higher toxicity burden.

The overall RCCEP incidence observed in our study, 33.3% in Group A and 25.0% in Group B, was numerically lower than the historically high incidence reported with camrelizumab monotherapy. In the multicenter phase II study by Wang et al., RCCEP occurred in 145 of 217 camrelizumab-treated patients, corresponding to an incidence of 66.8%, and all events were grade 1 or 2 (10). In addition, a 16-case clinical series and literature review summarized that RCCEP incidence with camrelizumab monotherapy reached 77.1% in one series and 97.3% in classical Hodgkin lymphoma, again with predominantly low-grade events (13). The results from two single-arm, phase 1 trials in nasopharyngeal carcinoma also reported an RCCEP incidence of 88% with camrelizumab alone (22). This interpretation is also supported by historical data from camrelizumab-plus-chemotherapy trials. In the ESCORT-1st trial in advanced or metastatic ESCC, RCCEP was reported in 238 of 298 patients (79.9%) receiving camrelizumab plus chemotherapy (23). In the CameL trial of chemotherapy-naive advanced nonsquamous NSCLC, grade 1 or 2 RCCEP occurred in 154 of 205 patients (75.1%), and grade 3 RCCEP occurred in 4 patients (2.0%) in the camrelizumab-plus-chemotherapy group (24).

Our findings are also directionally consistent with prior evidence suggesting that antiangiogenic strategies may reduce RCCEP risk. In the pooled analysis, RCCEP occurred in 23.6% of patients treated with camrelizumab plus apatinib, compared with 77.0% with camrelizumab monotherapy and 67.8% with camrelizumab plus chemotherapy (15). Xu et al. showed that among patients with advanced or metastatic hepatocellular carcinoma, gastric cancer, or gastroesophageal junction cancer, RCCEP occurred in 12.1% (4/33) of those treated with camrelizumab 200 mg every 2 weeks plus apatinib 250 mg once daily, while no RCCEP was reported in those receiving camrelizumab combined with apatinib 500 mg once daily (25). Similarly, in a phase I/II study of patients with EGFR/ALK wild-type advanced nonsquamous NSCLC, the incidence of RCCEP was 15.6% (15/96) with camrelizumab 200 mg every 2 weeks plus apatinib 250 mg once daily (26). Likewise, in a retrospective NSCLC study, camrelizumab combined with an antiangiogenic agent was independently associated with a lower risk of RCCEP, with an OR = 0.188 (24). Although our trial did not use a classical VEGF/VEGFR inhibitor, the RCCEP incidence in Group B was numerically close to that reported with camrelizumab plus apatinib and lower than the pooled incidence reported for camrelizumab plus chemotherapy, which is compatible with the hypothesis that thalidomide may exert a preventive effect through an antiangiogenic mechanism.

Comparison with the previously published thalidomide prophylaxis study is particularly relevant. In the prospective study by Song et al., RCCEP occurred in 2 of 9 patients (22.2%) in the thalidomide group versus 8 of 10 patients (80.0%) in the camrelizumab-alone group, and the median onset time of RCCEP was 5 weeks and 4 weeks, respectively (18). In our study, the overall RCCEP incidence in Group B was 25.0%, which was numerically close to the thalidomide-treated cohort reported by Song et al., whereas Group A still remained below most monotherapy-based historical series (10, 15). More recently, a multicenter, randomized, placebo-controlled phase II study in advanced NSCLC provided direct controlled evidence for thalidomide prophylaxis, with RCCEP occurring in 32.1% of patients receiving thalidomide compared with 64.2% receiving placebo (P = 0.0010) (27). This finding supports the rationale for thalidomide prophylaxis, whereas the present study specifically addresses dose selection between two active thalidomide regimens rather than the efficacy of thalidomide versus no prophylaxis.

Regarding dose effect, Group B had numerically lower overall RCCEP incidence, lower week 9 RCCEP incidence, and shorter RCCEP duration than Group A. The 8.3-percentage-point difference in overall RCCEP incidence exceeded the prespecified 5-percentage-point incidence-based selection threshold, and the 100 mg/day regimen therefore met the prespecified criterion for selection as the more promising dose. The selection rule was based on overall RCCEP incidence, and no prespecified safety-based dose-selection criterion was used. However, this study was not designed or powered to demonstrate statistically significant superiority between the two dose groups, and the numerical efficacy findings should therefore be interpreted as exploratory. This numerical advantage of the 100 mg/day dose was accompanied by a higher incidence of grade 3 or higher TEAEs and thalidomide-related AEs. Treatment-related hematologic toxicities, including neutrophil count decreased, white blood cell count decreased, and anemia, were more common in Group B. SAEs were also numerically more frequent in Group B, although the between-group difference was small. Thus, the potential efficacy advantage of the 100 mg/day dose should be interpreted together with its higher toxicity burden. Previous studies have shown that RCCEP associated with camrelizumab is usually low grade, with all RCCEP events reported as grade 1 or 2 in a multicenter phase II study of advanced hepatocellular carcinoma (10). In addition, in the prospective study by Song et al., thalidomide prophylaxis was associated with mild adverse events and no treatment-associated interruption (18).

Immune-related adverse events occurring during immune checkpoint inhibition may also have potential prognostic implications. Emerging evidence suggests that some low-grade cutaneous and endocrine immune-related adverse events may be associated with improved treatment outcomes, although their value as on-treatment prognostic markers remain incompletely established and may vary across tumor types (28).

The QoL findings in our study provide additional context for this risk-benefit balance. In the PRO-evaluable population, Group B showed numerically more favorable changes in global health status/QoL and selected functional domains than Group A. Although exploratory, this observation is potentially meaningful because cutaneous immune-related adverse events are clinically relevant toxicities that may adversely affect patients’ quality of life (29). Recent oncodermatology data further showed that dermatologic immune-related adverse events negatively affect QoL, that dermatologic intervention can improve QoL while facilitating ongoing oncologic treatment, and that dermatology-specific and oncology-oriented QoL instruments may capture different aspects of this burden (30).

Several limitations should be acknowledged. First, this randomized phase II selection trial compared two active thalidomide dose levels and did not include a placebo or no-thalidomide control group. Therefore, the study was designed to select the more promising dose rather than to establish the efficacy of thalidomide prophylaxis against an untreated control. Second, the sample size was relatively small and was determined for an exploratory phase II selection design; therefore, the findings require confirmation in larger, adequately powered studies. Third, the study included both ESCC and NSCLC, and heterogeneity in tumor type and chemotherapy backbone may have influenced baseline RCCEP risk. Fourth, oncologic outcomes, including objective response, disease control, camrelizumab interruption, and survival, were not systematically collected for this analysis, and no mechanistic biomarkers were evaluated; therefore, the study cannot determine whether thalidomide prophylaxis or RCCEP prevention affected antitumor efficacy or treatment continuity. In addition, the primary endpoint had no prespecified fixed risk window or final-analysis trigger, and 13 patients remained on treatment at the data cutoff; therefore, subsequent RCCEP events could alter the overall incidence and potentially the dose-selection result. Some RCCEP episodes were also unresolved at the cutoff, and their duration was calculated using the last available follow-up date. RCCEP assessment was open-label and not centrally adjudicated; therefore, interobserver variability across centers cannot be excluded. Finally, the QoL analysis was based on a limited evaluable population and should be interpreted cautiously. Larger, prospectively powered studies are warranted to further evaluate thalidomide dose selection, identify patients most likely to benefit from prophylaxis, and determine whether RCCEP prevention translates into improved treatment experience and continuity.

In conclusion, thalidomide 100 mg/day met the prespecified incidence-based dose-selection criterion and was selected as the more promising dose for further evaluation. However, the study did not establish statistical superiority or an optimal benefit-risk profile, and the 100 mg/day dose was associated with a higher incidence of grade 3 or higher toxicity. Further controlled studies are warranted.

## Data Availability

The data supporting the findings of this study are available from the corresponding author upon reasonable request. The data are not publicly available due to patient confidentiality and applicable ethical restrictions.
