# Supplementary material for: Thalidomide dose selection for prevention of camrelizumab-induced reactive cutaneous capillary endothelial proliferation: a multicenter, randomized phase II trial
Source: Front Immunol. 2026 Oct 16;17:1917046. doi: 10.3389/fimmu.2026.1917046 (PMC13719562; doi:10.3389/fimmu.2026.1917046)
Supplement: Supplementary file 1 [file Table1.docx]

Table S1. RCCEP characteristics in patients who developed RCCEP.

|  | Group A  (N= 12) | Group B  (N=9) |
| --- | --- | --- |
| RCCEP lesion sites, n (%) |  |  |
| Head and face | 7 (58.3) | 3 (33.3) |
| Neck | 3 (25.0) | 5 (55.6) |
| Trunk | 10 (83.3) | 6 (66.7) |
| Extremities | 5 (41.7) | 2 (22.2) |
| Oral mucosa | 0 | 0 |
| Eyelid | 0 | 1 (11.1) |
| Other | 0 | 2 (22.2) |
| Morphologic characteristics of RCCEP, n (%) |  |  |
| Red nevus-like | 9 (75.0) | 6 (66.7) |
| Pearl-like | 3 (25.0) | 1 (11.1) |
| Mulberry-like | 0 | 0 |
| Patch-like | 0 | 1 (11.1) |
| Tumor-like | 1 (8.3) | 0 |
| Others | 0 | 1 (11.1) |
| unknown | 1 (8.3) | 1 (11.1) |
| Bleeding status, n (%) |  |  |
| Yes | 2 (16.7) | 1 (11.1) |
| No | 10 (83.3) | 8 (88.9) |
| RCCEP lesion count categories, n (%) |  |  |
| ＜10 | 7 (58.3) | 5 (55.6) |
| 10-19 | 2 (16.7) | 1 (11.1) |
| 20-29 | 2 (16.7) | 2 (22.2) |
| 30-39 | 1 (8.3) | 0 |
| 40-49 | 0 | 0 |
| ≥50 | 0 | 1 (11.1) |
| Maximum diameter, (mm) |  |  |
| Median, (IQR) | 4.5 (3.5, 6.0) | 3.5 (2.0, 5.0) |

RCCEP, reactive cutaneous capillary endothelial proliferation; IQR, interquartile range
