# Supplementary material for: Thalidomide dose selection for prevention of camrelizumab-induced reactive cutaneous capillary endothelial proliferation: a multicenter, randomized phase II trial
Source: Front Immunol. 2026 Oct 16;17:1917046. doi: 10.3389/fimmu.2026.1917046 (PMC13719562; doi:10.3389/fimmu.2026.1917046)
Supplement: Supplementary file 2 [file Table2.docx]

Table S2. RCCEP characteristics in patients who developed RCCEP in the ESCC subgroup.

|  | Group A  (N= 6) | Group B  (N=4) |
| --- | --- | --- |
| RCCEP lesion sites, n (%) |  |  |
| Head and face | 5 (83.3) | 2 (50.0) |
| Neck | 1 (16.7) | 2 (50.0) |
| Trunk | 4 (66.7) | 3 (75.0) |
| Extremities | 3 (50.0) | 1 (25.0) |
| Oral mucosa | 0 | 0 |
| Eyelid | 0 | 1 (25.0) |
| Other | 0 | 1 (25.0) |
| Morphologic characteristics of RCCEP, n (%) |  |  |
| Red nevus-like | 3 (50.0) | 3 (75.0) |
| Pearl-like | 3 (50.0) | 0 |
| Mulberry-like | 0 | 0 |
| Patch-like | 0 | 0 |
| Tumor-like | 0 | 0 |
| Others | 0 | 1 (25.0) |
| unknown | 1 (16.7) | 0 |
| Bleeding status, n (%) |  |  |
| Yes | 2 (33.3) | 0 |
| No | 4 (66.7) | 4 (100.0) |
| RCCEP lesion count categories, n (%) |  |  |
| ＜10 | 5 (83.3) | 3 (75.0) |
| 10-19 | 1 (16.7) | 1 (25.0) |
| 20-29 | 0 | 0 |
| 30-39 | 0 | 0 |
| 40-49 | 0 | 0 |
| ≥50 | 0 | 0 |
| Number of RCCEP lesions |  |  |
| Median (IQR) | 5.0 (5.0, 5.0) | 5.0 (5.0, 10.0) |
| Maximum diameter, (mm) |  |  |
| Median, (IQR) | 5.0 (4.0, 7.0) | 2.0 (2.0, 2.5) |

RCCEP, reactive cutaneous capillary endothelial proliferation; IQR, interquartile range
