# Supplementary material for: Thalidomide dose selection for prevention of camrelizumab-induced reactive cutaneous capillary endothelial proliferation: a multicenter, randomized phase II trial
Source: Front Immunol. 2026 Oct 16;17:1917046. doi: 10.3389/fimmu.2026.1917046 (PMC13719562; doi:10.3389/fimmu.2026.1917046)
Supplement: Supplementary file 3 [file Table3.docx]

Table S3. RCCEP characteristics in patients who developed RCCEP in the NSCLCsubgroup.

|  | Group A  (N= 6) | Group B  (N=5) |
| --- | --- | --- |
| RCCEP lesion sites, n (%) |  |  |
| Head and face | 2 (33.3) | 1 (20.0) |
| Neck | 2 (33.3) | 3 (60.0) |
| Trunk | 6 (100.0) | 3 (60.0) |
| Extremities | 2 (33.3) | 1 (20.0) |
| Oral mucosa | 0 | 0 |
| Eyelid | 0 | 0 |
| Other | 0 | 1 (20.0) |
| Morphologic characteristics of RCCEP, n (%) |  |  |
| Red nevus-like | 6 (100.0) | 3 (60.0) |
| Pearl-like | 0 | 1 (20.0) |
| Mulberry-like | 0 | 0 |
| Patch-like | 0 | 1 (20.0) |
| Tumor-like | 1 (16.7) | 0 |
| unknown | 0 | 1 (20.0) |
| Bleeding status, n (%) |  |  |
| Yes | 0 | 1 (20.0) |
| No | 6 (100.0) | 4 (80.0) |
| RCCEP lesion count categories, n (%) |  |  |
| ＜10 | 2 (33.3) | 2 (40.0) |
| 10-19 | 1 (16.7) | 0 (0) |
| 20-29 | 2 (33.3) | 2 (40.0) |
| 30-39 | 1 (16.7) | 0 |
| 40-49 | 0 | 0 |
| ≥50 | 0 | 1 (20.0) |
| Number of RCCEP lesions |  |  |
| Median (IQR) | 15.0 (5.0, 25.0) | 25.0 (5.0, 25.0) |
| Maximum diameter, (mm) |  |  |
| Median, (IQR) | 4.0 (3.0, 5.2) | 5.0 (4.5, 5.0) |

RCCEP, reactive cutaneous capillary endothelial proliferation; IQR, interquartile range
